# Modified 30-second Sit to Stand test predicts falls in a cohort of institutionalized older veterans

**DOI:** 10.1371/journal.pone.0176946

**Published:** 2017-05-02

**Authors:** Eva V. Applebaum, Dominic Breton, Zhuo Wei Feng, An-Tchi Ta, Kayley Walsh, Kathleen Chassé, Shawn M. Robbins

**Affiliations:** 1 School of Physical and Occupational Therapy, McGill University, Montreal, QC, Canada; 2 Ste Anne’s Hospital, Sainte-Anne-de-Bellevue, QC, Canada; 3 Centre for Interdisciplinary Research in Rehabilitation; Constance Lethbridge Rehabilitation, Montreal, QC, Canada; West Chester University of Pennsylvania, UNITED STATES

## Abstract

Physical function performance tests, including sit to stand tests and Timed Up and Go, assess the functional capacity of older adults. Their ability to predict falls warrants further investigation. The objective was to determine if a modified 30-second Sit to Stand test that allowed upper extremity use and Timed Up and Go test predicted falls in institutionalized Veterans. Fifty-three older adult Veterans (mean age = 91 years, 49 men) residing in a long-term care hospital completed modified 30-second Sit to Stand and Timed Up and Go tests. The number of falls over one year was collected. The ability of modified 30-second Sit to Stand or Timed Up and Go to predict if participants had fallen was examined using logistic regression. The ability of these tests to predict the number of falls was examined using negative binomial regression. Both analyses controlled for age, history of falls, cognition, and comorbidities. The modified 30-second Sit to Stand was significantly (p < 0.05) related to if participants fell (odds ratio = 0.75, 95% confidence interval = 0.58, 0.97) and the number of falls (incidence rate ratio = 0.82, 95% confidence interval = 0.68, 0.98); decreased repetitions were associated with increased number of falls. Timed Up and Go was not significantly (p > 0.05) related to if participants fell (odds ratio = 1.03, 95% confidence interval = 0.96, 1.10) or the number of falls (incidence rate ratio = 1.01, 95% confidence interval = 0.98, 1.05). The modified 30-second Sit to Stand that allowed upper extremity use offers an alternative method to screen for fall risk in older adults in long-term care.

## Introduction

Falls are the leading cause of accidental death and nonfatal injury in older adults [1,2]. In short- and long-term care hospital settings, the prevalence of falls varies between 1 to 9 per 1000 beds per day with 30–50% of these falls causing injury [3,4]. Previous studies have investigated fall risk factors in order to identify older adults at risk of falling so that preventative interventions can be applied.

Published guidelines suggest that older adults should be assessed for fall risk annually [5]. Screening procedures include identification of risk factors and scores on various functional performance tests. Some risk factors for falls in older adults that have been reported include history of falls, over 80 years of age, comorbidities, generalized deconditioning, muscle weakness, and impaired cognition [6–8]. Furthermore, a history of falls (odds ratio = 3.06), walking aid use (odds ratio = 2.08), and moderate disability (odds ratio = 2.08) are the strongest factors associated with falling in long-term care residents [9]. The ability to identify these risk factors can guide clinicians to which older adults should receive preventative interventions.

A substantial portion of falls in institutionalized older adults occur during transfers and walking and evaluating these activities during fall screening is important [10]. A variety of functional performance tests assess these activities and their ability to predict falls have been examined. The Timed Up and Go (TUG) is a timed test that requires participants to rise from a chair, walk three meters, and return to the chair [11]. Studies reported that TUG can differentiate between fallers and non-fallers retrospectively, but other studies report that it is not related to falls measured prospectively in older adults [12–15]. The reason for this discrepancy is not apparent, but perhaps the TUG is not a sensitive indicator of deterioration in strength and balance. Other test options include variations of Sit to Stand (STS) tests. The Five-Time STS, which records the amount of time to complete five STSs, significantly predicted (relative risk = 1.74) recurrent falls in community living older adults [16]. Similarly, a retrospective study concluded that an inability to complete the Five-Time STS was a significant predictor of falls (odds ratio = 4.22) over three years [17]. Thus, functional performance tests can be utilized to screen older adults at risk of falling.

Despite the benefit of STS tests to measure physical function and potentially predict falls, they have imitations. Repetition based STS protocols (e.g. Five-Time STS) have a restricted capacity to assess a wide variation in ability, which is relevant within a geriatric institutionalized setting [18]. Specifically, some older adults cannot complete the five trials and are not assigned a score, hence limiting the test’s utility in older adults with moderate to severe mobility limitations. Consequently, for this population the literature favors timed based protocols such as the 30-Second Sit to Stand Test (30STS), where the number of repetitions within 30 seconds is recorded [19]. The 30STS is more easily completed and a score will be given regardless of the individual’s ability to complete the test. An additional modification to accommodate older adults with lower levels of physical function includes upper extremity use with armrests. Without the use of their upper extremities, institutionalized older adults are often unable to complete a single repetition. This modification would reflect a more realistic method of standing up and would promote better “ecological validity” [20].

Although these suggestions have been made, the ability of this modified 30STS (m30STS) to predict falls has not been examined. Furthermore, research examining the relationship between TUG and fall risk is conflicting and requires additional investigation. Therefore, the objective was to determine the ability of the m30STS, that permits upper extremity use, and TUG to predict if older adults will fall and to predict the number of falls they will sustain over a one year period in an institutionalized geriatric setting. It was hypothesized that the m30STS, but not the TUG, would be related to falls over one year. This is the first known study to evaluate the relationship between falls and m30STS with upper extremity use. Recently, the m30STS was found to have good test-retest reliability [intraclass correlation coefficient (ICC) = 0.84] and convergent validity with the TUG (r = -0.62) in older adults [21].

## Materials and methods

### Design and participants

This longitudinal, observational cohort study was conducted from 2014 to 2015 at St-Anne’s Veterans Hospital (Quebec, Canada), a hospital that specializes in the long-term care of Veterans. A convenience sample of older adults was recruited by physiotherapists between May and June of 2014. Participants were included if they were: 1) at least 65 years of age, 2) reside at the hospital, and 3) physically and mentally capable of completing the assessments. Exclusion criteria included: 1) severe cognitive impairments (Mini-Mental State Exam < 10) [22], 2) restrictions on weight-bearing status (e.g. following recent hip fracture), and 3) unable to follow verbal commands. Participants were also able to ambulate short distances independently with or without assistive devices in order that they could complete the TUG.

Initial baseline data were used in a concurrent study to examine the psychometric properties of the m30STS [21]. The current analysis was a continuation of this previous study and included one year follow-up data. The sample size calculation was based on the previous study’s analysis [21]. In total, baseline data for 62 participants were available for the current study. The Centre for Interdisciplinary Research in Rehabilitation of Greater Montreal ethics board and Veteran Affairs Canada approved the study. Informed, written consent was obtained from each participant.

### Demographic and control variables

In order to describe the study sample, demographic variables and other measures were collected from medical charts at baseline including age, sex, body mass index, history of falls one year prior to baseline testing, use of a walking aid, cognition, and comorbidities. Comorbidities were measured using the Charlson Comorbidity Index [23]. Cognition was measured using the Mini-Mental State Exam [22]. In addition, some of these measures were entered as control variables in the primary analyses since they have previously demonstrated an association with falling including age, history of falls, cognition, and comorbidities [6–9,24]. Sex was not entered as a control variable due to the low proportion of women. Body mass index was not entered as a control variable since there is no evidence that it is a risk factor for falls in older adults residing in long-term care.

### Independent variables

#### Modified 30 second sit to stand

The m30STS was collected at baseline using consistent procedures for all participants. Prior to each test, clear and simple instructions were given orally and were followed by a standardized demonstration. Participants were allowed one practice trial before the actual measurement. They were permitted to use their upper extremities and armrests. Participants were seated in a standard chair with armrests (seat height 17 inch, seat width 18 inch). The same chair was used for all participants. They were instructed to sit in the middle of the chair, back straight, feet approximately shoulder-width apart, and placed on the floor at an angle slightly back from the knees with one foot slightly in front of the other to help maintain balance when standing [25]. Instructions to participants were: “When I say ‘1, 2, 3, go’, I want you to stand up and then sit down again. You can use your hands to help you stand if you need to. Try to stand and sit back down as many times as possible while I time you for 30 seconds.” Participants were encouraged to continue to sit and stand throughout the test. The number of STS repetitions was recorded which represented the units for this measure. Our previous study demonstrated excellent test-retest reliability (ICC = 0.84) for the m30STS in this sample [21].

#### Timed Up and GO Test

TUG was also collected using a standard protocol [11]. Participants were allowed one practice trial before the actual measurement. They were seated in a standard chair with armrests (seat height 17 inch, seat width 18 inch). Standard instructions were: “When I say ‘1, 2, 3 go’, please stand up from the chair, walk [with your assistive device] at a comfortable pace to the line, return to the chair, and sit down.” The line was three meters from the chair, marked with tape, and testers confirmed that each participant was aware of the line. Participants were permitted to use their walking aid if they usually used one for ambulation. They were timed to the nearest 1/100 second using a digital stopwatch and the unit for TUG was seconds. Each participant completed two trials and the mean score was used for data analysis. Participants were allowed to rest as long as needed between trials. TUG has demonstrated excellent inter-rater reliability (ICC = 0.91 to 0.99) and test-retest reliability (ICC = 0.74 to 0.99) [15].

### Dependent variables: Fall status and number of falls

Fall status (faller vs. non-faller) and number of falls over a one year period following baseline testing were the dependent variables. This was recorded through a well-established and rigorous protocol that was implemented by the hospital. Falls were prospectively recorded each time a participant accidently came to the ground, the floor, or a lower level. The participant’s nurse immediately sent the fall information to the rehabilitation clinical coordinator. This person ensured that a follow up took place and recorded the fall in a database. The number of falls of each participant during the one year period after baseline was extracted from this database. Likewise, the history of falls one year prior to baseline testing was also recorded.

### Study procedures

Demographic information (age, sex, body mass index) and control variables (age, history of falls during the one year period before baseline testing, cognition, and comorbidities) were extracted from the participants’ charts. Participants then completed the m30STS followed by the TUG at baseline. Participants were allowed to rest as long as needed between these tests in order to limit any potential fatigue effects. Researchers then collected the number of falls during the one year period following baseline testing. A review of the participants’ charts was also completed in order to extract other relevant data (e.g. absences from the hospital, deaths) that were used to describe the sample.

### Data analysis

Descriptive statistics were calculated for the demographic and study variables. Pearson correlation coefficients between continuous variables and point biserial correlation coefficients between dichotomous and continuous variables were calculated. Two-tailed t-tests examined the significance of each correlation coefficient. In order to examine if variables predicted both the risk of falling at least once (fallers vs. non-fallers) and the number of falls, separate analyses were conducted for these dependent variables. For the dichotomous dependent variable, specifically fall status (fallers vs. non-fallers), a logistic regression examined the ability of the m30STS (number of repetitions) and TUG (time in seconds) to explain the variance in falls. Unadjusted analyses were first conducted where either m30STS or TUG were the lone independent variable in the model. For adjusted analyses, control variables (age, history of falls as a dichotomous variable, cognition, and comorbidities) were entered in the first step of the analysis. The m30STS scores were entered in the final step. This analysis was repeated with the TUG scores entered in the final step. The m30STS and TUG were not entered into the same model because of concerns of multicollinearity since it was expected these variables would be highly correlated. Odds ratio with a 95% confidence interval (CI) was examined for each variable and a chi-square test was used to determine the overall model fit. Likewise, negative binomial regression analysis examined the ability of the m30STS and TUG to explain the variance in the number of falls over one year. Once again, both unadjusted and adjusted models were performed. Incidence rate ratios with 95% CI were examined for each variable and the likelihood ratio chi-square statistic examined overall model fit. To ensure that the results of the statistical tests were trustworthy, assumptions were examined for statistical analysis. For logistic regression this involved examining linearity, multicollinearity, assumptions of independence, and potential interactions. For negative binomial regression analysis, this involved examining linear relationships, residuals, mutlicollinearity, and potential interactions [26]. These analyses were completed using SPSS (version 20.0).

Additionally, secondary analyses were completed. Receiver Operating Characteristic (ROC) curves further examined the ability of m30STS and TUG to prospectively classify participants as fallers or non-fallers [27]. Since scale direction for m30STS (higher scores represent higher physical function) and TUG (higher scores represent lower physical function) are opposite, m30STS values were transformed by taking the inverse prior to ROC analysis. Area under the curve (AUC) values, which provide an indication of the overall diagnostic accuracy of a test, with 95% CI were calculated. The optimal cut point that produced the highest “diagnostic” accuracy, and its associated sensitivity and specificity were determined for both m30STS and TUG. In addition, AUC values were compared between these measures using a previously described bootstrap procedure with a 1000 samples [27]. For each of the bootstrap samples, AUC values were calculated for m30STS and TUG and the difference in these AUC values were determined. The mean and 95% CI of these differences were then calculated. All ROC analyses were unadjusted (i.e. did not consider control variables) and were completed with custom software written in Matlab (version 7.14).

## Results

Eighty potential participants were considered for the study. Nine potential participants declined to participate. One potential participant was excluded due to severe cognitive impairment (Mini-Mental State Examination score < 10), and one was excluded because he was unable to ambulate independently (with or without an assistive device). Seven suggested participants were not included because they were unable to attend the data collection session due to other commitments. Sixty-two participants completed baseline testing but nine participants were deceased during the one year period after baseline. Therefore, 53 participants completed the study and were analyzed. Only participants with complete data were included in the final analyses. Absences from baseline to the one year follow-up ranged from 0 to 22 days, with the exception of one participant who was discharged home resulting in 52 days of absence. Participants were still included in the analyses despite these absences in order to maximize the study sample.

Baseline descriptive statistics for the 53 participants (49 men, 4 women) are shown in Table 1. The participants were Veterans which accounts for the large proportion of men. The mean Charlson Comorbidity Index scores showed that the sample had numerous or severe comorbidities (Table 1). In order to complete the TUG, 50 of the 53 participants used an assistive device including a two-wheeled walker (n = 12) or four-wheeled walker (n = 38). Twenty-one out of the 53 participants had a history of falling during the one year period prior to baseline testing. Thirty-two out of the 53 participants fell in the one year period after baseline testing. Fifteen participants fell once, three participants fell twice, four participants fell three times, four participants fell four times, two participants fell five times, and four participants fell six times.

**Table 1:** Characteristics of the study participants (n = 53).

*Variable*	*Mean (SD)*	*Minimum, Maximum*
Age (years)	91 (4)	82, 98
Body Mass Index (kg/m^2^)	26.10 (5.12)	17.90, 48.09
Mini-Mental State Examination	23 (5)	12, 30
CCI (i)	8 (2)	5, 12
m30STS (number of repetitions)	5 (3)	1, 16
TUG (seconds)	26.20 (9.20)	7.91, 45.64
Falls in previous year	1 (2)	0, 9
Number of falls^a^	2 (2)	0, 6

CCI (i) = Charlson Comorbidity Index; m30STS = modified 30 second sit to stand test; TUG = Timed Up and Go Test.

^a^Number of falls over one year after baseline testing.

Correlations coefficients between each variable are shown in Table 2. A strong correlation was found between TUG and m30STS (r = -0.63, p < 0.01). Moderate correlations were found between m30STS and number of falls (r = -0.30, p = 0.03), and between Mini-Mental State Examination scores and history of falls (r = -0.37, p < 0.01). A weak correlation was found between age and number of falls in the one year period after baseline testing (r = 0.29, p = 0.03). Other correlations were weak and statistically non-significant.

**Table 2:** Pearson/point-biserial correlation coefficients (p value) between study variables.

Variables	Number of falls^a^	Fall in previous year^b^	Age	MMSE	CCI (i)	m30STS	TUG
Number of falls^a^	−	0.18 (0.20)	0.29 (0.03)	-0.15 (0.28)	0.08 (0.58)	-0.30 (0.03)	0.14 (0.34)
Fall in previous year^b^		−	0.17 (0.23)	-0.37 (<0.01)	-0.07 (0.60)	-0.05 (0.71)	0.14 (0.31)
Age			−	-0.22 (0.11)	0.09 (0.52)	-0.08 (0.56)	0.19 (0.18)
MMSE				−	0.09 (0.51)	-0.03 (0.85)	-0.05 (0.73)
CCI (i)					−	-0.21 (0.14)	0.08 (0.59)
m30STS						−	-0.63 (<0.01)
TUG							−

MMSE = Mini-Mental State Examination; CCI (i) = Charlson Comorbidity Index; m30STS = modified 30 second sit to stand test; TUG = Timed Up and Go Test.

^a^Number of falls over one year after baseline testing.

^b^Analyzed as dichotomous data (yes or no fall in the one year prior to testing).

For the logistic regression with no control variables, m30STS had a borderline significant relationship (odds ratio = 0.78, 95% CI = 0.60, 1.01; p = 0.06) with fall status (e.g. faller vs. non faller) over one year. Results of the logistic regression analysis with control variables are shown in Table 3. After controlling for age, history of falls, cognition, and comorbidities, m30STS significantly (χ^2^ = 5.72, p = 0.02) explained the variance in fall status over one year. Only the odds ratio for the m30STS was significant (Table 3) and showed that for every decrease in one STS repetition during testing, there was a decreased risk of falling by 0.75 times. The overall model, which included the m30STS and control variables, was not significant (χ^2^ = 9.84, p = 0.08). When the TUG was entered as the lone variable, it was not significantly related to fall status (odds ratio = 1.03, 95% CI = 0.97, 1.10; p = 0.35). After entering the control variables, TUG was also not significantly (χ^2^ = 0.64, p = 0.43) related to fall status (Table 3). The overall model was also not significant (χ^2^ = 4.76, p = 0.45). The assumptions for logistic regression were examined and there were no issues with linearity, independence of errors, multicollinearity, or interactions.

**Table 3:** Logistic regression analysis of fall status (fallers vs. non-fallers) over one year after baseline testing.

Model	Independent Variables	OR	95% CI for OR	p value^a^
1				
	Fall in previous year^b^	1.07	0.29, 3.97	0.92
	Age	1.11	0.91, 1.36	0.29
	MMSE	0.95	0.83, 1.08	0.39
	CCI (i)	0.70	0.46, 1.07	0.10
	m30STS^c^	0.75	0.58, 0.97	0.03
2				
	Fall in previous year^b^	1.02	0.29, 3.60	0.96
	Age	1.10	0.92, 1.32	0.31
	MMSE	0.95	0.84, 1.08	0.46
	CCI (i)	0.78	0.53, 1.15	0.21
	TUG^c^	1.03	0.96, 1.10	0.43

OR = odds ratio; CI = confidence interval; MMSE = Mini-Mental State Examination; CCI (i) = Charlson Comorbidity Index; m30STS = modified 30 second sit to stand test; TUG = Timed Up and Go Test.

^a^ p value is for the Wald χ^2^ statistic.

^b^ Analyzed as dichotomous data (yes or no fall in the one year prior to testing).

^c^ Units for m30STS was number of repetitions, and time in seconds for TUG.

For the negative binomial regression analysis with no control variables, m30STS had a significant relationship (incidence rate ratio = 0.81, 95% CI = 0.67, 0.97; p = 0.02) with the number of falls over one year. Results of the negative binomial regression analysis with control variables are shown in Table 4. After controlling for age, history of falls, comorbidities, and cognition, m30STS significantly (Wald χ^2^ = 4.57, p = 0.03) explained the variance in the number of falls over one year. An increase in the number of m30STS repetitions was associated with a decrease in the number of falls. The overall model was not significant (χ^2^ = 10.55, p = 0.06). Furthermore, tests on individual regression coefficients indicated that only m30STS was significantly related to the number of falls (Table 4). Comparatively, TUG alone did not significantly explain the variance in the number of falls over one year (incidence rate ratio = 1.02, 95% CI = 0.98, 1.05; p = 0.39). If control variables were entered in first step, TUG also did not significantly (Wald χ^2^ = 0.38, p = 0.54) explain the variance in the number of falls (Table 4) and the overall model was not significant (χ^2^ = 5.82, p = 0.32). The assumptions for negative binomial regression analysis were examined and there were no issues with linearity, multicollinearity, or interactions.

**Table 4:** Negative binomial regression analysis of the number of falls over one year after baseline testing.

Model	Independent Variables	IRR	95% CI for IRR	p value^a^
1				
	Fall in previous year^b^	1.33	0.57, 3.06	0.51
	Age	1.09	0.97, 1.21	0.15
	MMSE	0.97	0.90, 1.06	0.51
	CCI (i)	0.99	0.80, 1.24	0.96
	m30STS^c^	0.82	0.68, 0.98	0.03
2				
	Fall in previous year^b^	1.38	0.60, 3.15	0.45
	Age	1.10	0.98, 1.22	0.10
	MMSE	0.98	0.91, 1.06	0.64
	CCI (i)	1.01	0.81, 1.26	0.90
	TUG^c^	1.01	0.98, 1.05	0.54

^a^ p values are for the Wald χ^2^ statistic for the regression coefficient.

^b^ Analyzed as dichotomous data (yes or no fall in the one year prior to testing).

^c^ Units for m30STS was number of repetitions, and time in seconds for TUG.

IRR = incidence rate ratio; CI = confidence interval; MMSE = Mini-Mental State Examination; CCI (i) = Charlson Comorbidity Index; m30STS = modified 30 second sit to stand test; TUG = Timed Up and Go Test

**Fig 1:**
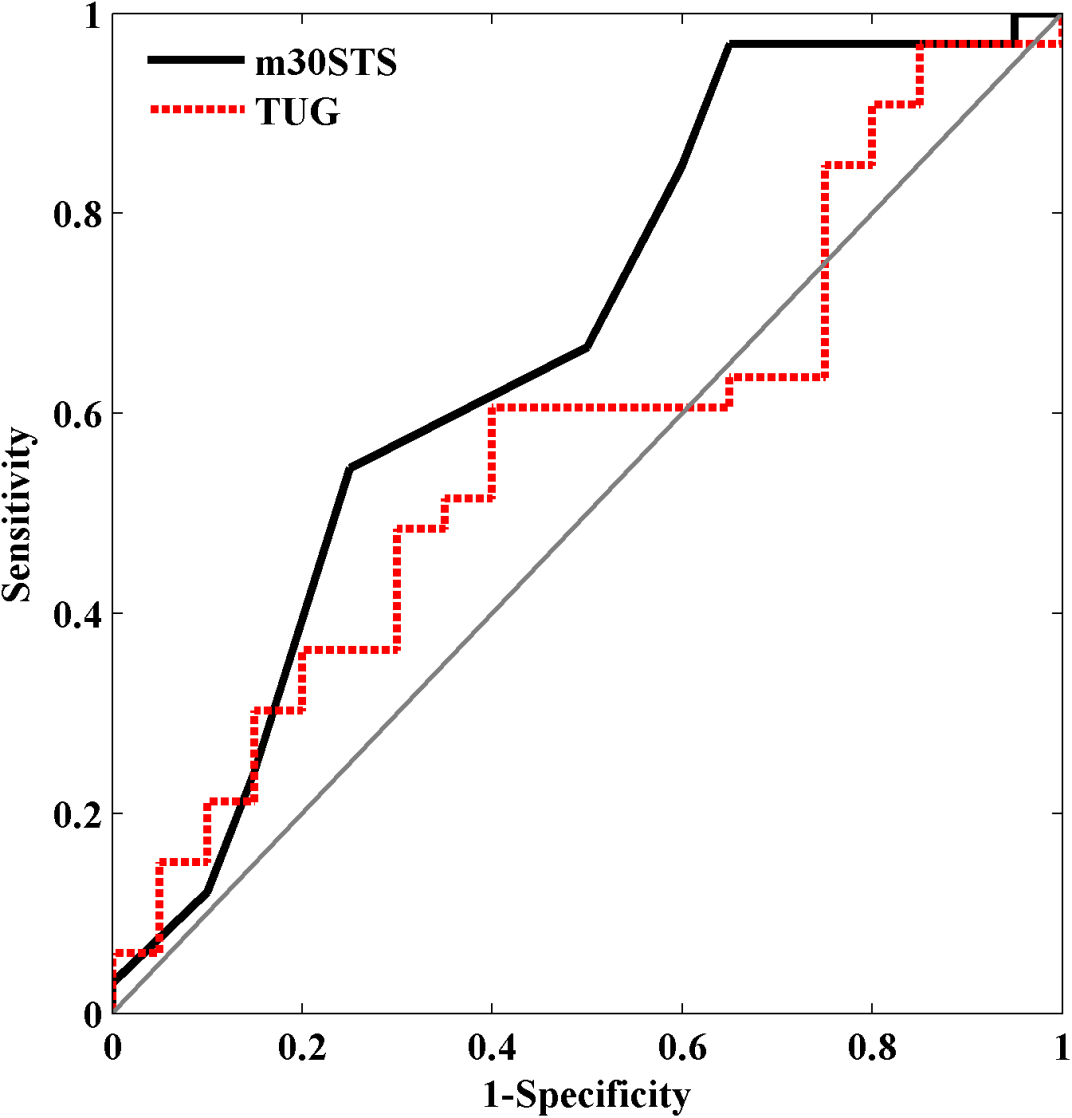
Receiver Operating Characteristic (ROC) curves for the modified 30-second Sit to Stand (m30STS; thick, solid, black line) and Timed Up and Go (TUG; thick, dashed, red line). The thin, solid, grey line represents if a random variable was used to predict fall status, which would have an area under the curve of 0.50.

ROC curves are provided in Fig 1. AUC for m30STS was 0.67 (95% CI = 0.48, 0.81) which was statistically significant (p = 0.04) indicating it was more effective at predicting fall risk than a random predictor (AUC = 0.50). The optimal cut point for m30STS was 7 repetitions, which produced a sensitivity of 0.97 and specificity of 0.35. AUC for TUG was 0.57 (95% CI = 0.40, 0.73) which was not statistically significant (p = 0.39). The optimal cut point for TUG was 14.2 seconds, which produced a sensitivity of 0.97 and specificity of 0.15. A comparison of m30STS and TUG AUC values using bootstrapping indicated the mean difference in AUC values was 0.10 (95% CI = -0.05, 0.26) which was not statistically significant (p = 0.19).

## Discussion

The m30STS has the ability to significantly explain variance in fall status (faller vs. non faller) and number of falls recorded prospectively over one year in older adults living in a long-term care hospital. An increase in STS repetitions was associated with fewer falls indicating that motor performance is important to consider when screening for fall risk. This modified version also provides a more realistic evaluation of STS performance by allowing upper extremity use and does not have the same potential for the assignment of “no score” as previous STS protocols (e.g. Five-Time STS). In contrast, TUG was not related to falls. Results support the m30STS as an alternative to TUG when measuring physical function and screening older adults for fall risk.

The results are supported by previous research. Although this is the first known study to evaluate the relationship between falls and m30STS with upper extremity use, previous articles have examined Five-Time STS. An inability to complete the Five-Time STS test was marginally associated with a greater risk of falling (odds ratio = 4.22, p = 0.09) over three years in older adults compared to those who required the shortest time to complete the test [17]. The Five-Time STS predicted recurrent falls (risk ratio = 1.74) in community dwelling older adults [16] and fall incidence (relative risk = 1.41) in older adult Veterans living in military communities [28]. Taking longer than 12 seconds on the Five-Time STS test predicted multiple falls (relative risk = 2.00) in community dwelling older adults [29]. In general, the TUG has demonstrated an inability to prospectively predict falls. A meta-analysis examining community dwelling older adults indicated the TUG was not a predictor of falls (odds ratio = 1.01) [30]. Another meta-analysis examining institutionalized and community dwelling older adults found there was an association between TUG and falls [31]. However, once analyses were adjusted for demographic or functional measures, TUG remained an independent predictor of falls in only two of five studies that examined institutionalized older adults [31]. Therefore, m30STS is more highly related to falls than TUG and should be considered when assessing older adults for fall risk.

Considering the current results and previous studies, STS tests have stronger relationships with fall outcomes than TUG but the reason for this finding is not clearly evident [29,30]. Researchers have stated that STS is an indicator of lower extremity strength which is important to consider since lower extremity weakness is a risk factor for falls and recurrent falls [32,33]. Perhaps repetitive STS tasks require greater muscle endurance and strength than TUG. In support of this hypothesis, various STS protocols have demonstrated moderate to strong correlations (r = 0.43 to 0.77) with lower extremity strength measures in older adults [18,33]. Also, the power required to complete one STS repetition was lower in older adults with a history of falling compared to no fall history highlighting the required strength to successfully complete a STS [34]. However, the current m30STS protocol permitted upper extremity use which would likely decrease the required lower extremity strength to complete the STS repetitions. Although it was not tested, it could be argued that the relationship between the m30STS and strength would be diminished compared to more traditional STS tests. How upper extremity use during STS tests impacts the relationship with falls is also not clear. The strength of the relationship between STS tests and falls are difficult to compare between studies due to differences in the testing protocol (e.g. Five-Times STS vs. 30STS) and statistical procedures (e.g. treating outcomes as continuous or dichotomous) [16,17,28,29]. Regardless, the m30STS has stronger relationships with prospective falls than TUG and research is needed to further explore potential explanations for this finding.

Despite the significant relationship between m30STS and falls, overall models were not significant. Analyses were likely underpowered for these models since the sample size calculation was performed for a different analysis. Alternatively, the high age of the current participants (mean age of 91) might have decreased the strength of the associations between control variables and falls. A similar ceiling effect has been recognized previously, specifically the association between risk factors and disease outcomes decreases with increasing age [35]. Also, history of falls was not significantly related to prospective falls despite previously showing a strong relationship [9]. The reason for this discrepancy is not clear. Finally, other important risk factors for falls should be considered in the future such as balance, executive function during dual tasks, and extrinsic environmental facts (e.g. cluttered environment) [36–38]. Nevertheless, m30STS was significantly related to falls and should be considered for inclusion in future fall prediction models for institutionalized older adults.

The unadjusted ROC analyses indicated that m30STS, but not TUG, could significantly “diagnose” or predict which participants would fall. However, there was no significant difference between AUC for m30STS and TUG. The optimal cut point for m30STS was 7 repetitions which was associated with high sensitivity (0.97), but low specificity (0.35). The high sensitivity, which was calculated from the inverse of m30STS scores, indicated that if participants completed more than 7 repetitions, then they were unlikely to fall. However, the low specificity indicated that less than 7 repetitions did not accurately predict fall status. A previous study determined the optimal cut point for 30STS was 15 repetitions and AUC was 0.79 [39]. This previous study was performed in community dwelling older adults with a mean age of 72 years, higher proportion of women, and lower fall rate [39]. These sample differences likely account for differences in optimal cut point and AUC.

Limitations include the participants resided in one hospital, were predominantly men, and had a mean age of 91 years. This limits the generalizability of the findings. Potential participants with severe cognitive and motor impairments were excluded because they were unlikely to complete the required tests and other measures should be used to screen fall risk in these individuals. Falls were prospectively recorded by the staff at the hospital if they witnessed the participant fall or if the participant was on the floor. Thus, it is possible that some participants fell but were able to get off the floor independently resulting in an underreporting of falls. Also, the context of falls, including the patient’s activity when they fell and the surrounding environment, was not available and would have provided a more complete picture.

## Conclusions

In conclusion, m30STS, but not TUG, had the ability to discriminate between fallers and non-fallers and was related to the number of falls recorded prospectively over one year in institutionalized older adults. Future research should account for additional fall risk factors and include a larger sample in order to draw stronger conclusions. Nevertheless, m30STS is an alternative, cost-effective, and time sensitive method for clinicians to assess physical function and screen for fall risk in older adults.

## Supporting information

S1 Dataset Dataset for the study sample. (XLSX)
